# Retraction: Modified CPT-TODIM method for evaluating the development level of digital inclusive finance under probabilistic hesitant fuzzy environment

**DOI:** 10.1371/journal.pone.0286616

**Published:** 2023-05-30

**Authors:** The PLOS ONE Editors

After this article was published, similarities were noted between this article and submissions by other research groups which call into question the validity and provenance of the reported results, and the adherence of this article to the PLOS Authorship policy. Further editorial assessment identified concerns regarding the integrity of the peer review process. In light of these issues, the *PLOS ONE* Editors retract this article [1].

YD did not agree with the retraction. WZ either did not respond directly or could not be reached.
